# The Morbid Anatomy of the Bowels, Liver, and Stomach

**Published:** 1829-01-01

**Authors:** 


### THE MORBID ANATOMY OF THE BOWELS, LIVER, AND STOMACH, ILLUSTRATED BY A SERIES OF PLATES FROM DRAWINGS AFTER NATURE, WITH EXPLANATORY LETTER-PRESS, AND A SUMMARY OF THE SYMPTOMS OF THE ACUTE AND CHRONIC AFFECTIONS OF THE ABOVE-NAMED ORGANS. By *John Armstrong*, M.D. &c.

[Fasciculus the Second.]

IN our last Number (page 347-58) we gave a full account of the first Fasciculus of Dr. Armstrong's splendid work. We now proceed to an analysis of the second, the plates of which are numerous and beautiful—indeed, too beautiful, the common and universal fault of all pathological representations.

The talented author observes, in the opening of the present Fasciculus, that Morbid Anatomy shews four prolific causes of disease (they are the diseases themselves)—inflammation, tubercle, fungus, and scirrhus. The effects of inflammation have been portrayed in our first article, to which we may refer.

"With respect to Tubercle, it hardly ever attacks the Stomach, except it be now and then seen in the Serous membrane, where the peritoneum has been studded, by that deposition, in points. But Fungus and Scirrhus are not uncommon in this country, sometimes seated about the Œsophagus, and sometimes about the Stomach, and not unfrequently, either the one or the other is found in both these parts in the same case. Simple Inflammation, however, by which is meant Inflammation uncombined with any of the above-mentioned peculiar Formations, sometimes attacks the Œsophagus, and may lead, like Fungus and Scirrhus, to Stricture there; though it must be confessed, that such an occurrence far more often depends on those adventitious Structures, than upon the thickening which results from simple Inflammation, and which is displayed in one of the drawings made from a recently fatal case. The very few cases of Stricture of the Œsophagus which I have seen to depend upon Inflammation alone were not seated about the Cardia, but higher up in that canal; whereas in those which have fallen under my observation as the effects of Fungus or Scirrhus, the Stricture was mostly in the vicinity of the Cardia. In three or four cases, I have known less or more obstruction to arise about the Pylorus, from the thickening of its cellular membrane through simple Inflammation; but what is usually called Stricture of the Pylorus depends upon the presence of Fungus or Scirrhus, and my own dissections would authorise me to conclude, that Fungus of the Cardia, Stomach, and Pylorus occurs quite as frequently as Scirrhus in those situations—a fact which has not, perhaps, been sufficiently attended to by authors who have expressly written on Morbid Anatomy.

"Both Fungus and Scirrhus seemingly attack almost every variety of structure; yet, from the attachment and derangement which each effects in the surrounding parts, during their growth and destruction, it is often difficult, and even impossible to fix upon the precise texture in which the disease had commenced. But indisputably the cellular connecting membrane of organs is the most common seat of both these extraneous productions, especially with reference to the Œsophagus and Stomach; though some minute examinations would induce me to infer, contrary to the opinion of some of my friends, that the true Fungus Encephaloides occasionally springs from a mucous surface, not only of these, but of other organs.

"The great alteration of Stucture, then, and consequently of office, in the Œsophagus and Stomach results from Inflammation, Fungus, or Scirrhus, and this remark is singularly applicable to what is called Stricture, which sometimes, however, has a truly spasmodic character in the Œsophagus, as shall be shown in the detail of the symptoms connected with those morbid appearances, which the plates of this Fasciculus are intended to elucidate." 50.

### STRICTURE OF THE ŒSOPHAGUS FROM SIMPLE INFLAMMATION.

This form of stricture is rare. It may take place either under the pharynx or near the cardia, and is preceded by signs of irritation of the mucous membrane of the stomach, such as flatulence, acidity, epigastric uneasiness, redness of the point and margin of the tongue, prominence of the papillæ.

"In instances of this sort, more or less redness is discoverable about the fauces and pharynx; for, though the irritation commonly begins in the inner lining of the Stomach, it is a striking fact, that Mucous membranes are apt to suffer secondarily toward their extremities. Hence the frequency of sympathetic Inflammation about the fauces and the pharynx of those whose Stomach is out of order from Mucous irritation; and this view of the subject is so important, that little impression can be made in Chronic Inflammation of those parts, unless the original remote disorder be constantly regarded in the plan of treatment. If the Inflammation arise under the pharynx, some degree of pain and impediment in swallowing exist there, with an increased secretion from the throat; and the increase or diminution of these symptoms mark the advancement or subsidence of the Inflammation, together with the declension or improvement of the general health. It must be borne in mind, that Chronic Inflammation about the epiglottis, rima glottidis, and pharynx, gives rise to great uneasiness in the act of deglutition; but such cases will be discriminated, first, by the hoarseness, peculiar cough, and the other signs which mark a laryngeal affection; and, secondly, by the sense of suffocation produced by every attempt to swallow any thing irritating the epiglottis, which then frequently performs its office imperfectly. During the progress of Stricture of the Œsophagus from Inflammation, it is accompanied by a sense of heat and uneasiness in the posterior part of the mouth, and also by difficulty of swallowing, a copious secretion of saliva, slight cough, and ultimately some degree of dyspnœa. If ulceration take place, almost all the symptoms are aggravated, and in the attacks of nausea or retching, a tenacious mucus is ejected, sometimes mixed with pus or blood. Ulceration of the Œsophagus occasionally proves fatal by excessive hemorrhage, and I have known the same event happen from Ulceration of the Stomach. But if the patient should not be thus suddenly carried off, great emaciation takes place, with fever of a hectic type, and a little before death, there is often feebleness, and even aberration of mind, as is seen in most instances where patients die from inanition." 62.

Spasmodic stricture of the œsophagus reveals its true character by the suddenness of its accession and retrocession. It is most common in nervous individuals, and hysterical females. In some dissections, Dr. Armstrong has found the cardia reddened, thickened, and softened, apparently from chronic inflammation, and on tracing the history, it appeared that the patients had had a sense of uneasiness and obstruction in that quarter during the passage of solids or fluids. This sense of obstruction, however, is very common where no organic change can be going on in the part, especially among females of nervous temperament.

### Stricture of the Cardia from Fungus or Scirrhus.

These two diseases cannot be distinguished by symptoms during life. It may be borne in mind, however, that scirrhus most frequently occurs after the age of 40 years—and that fungus may attack at any period of life. The progress of fungus may be expected to be more rapid than that of scirrhus—but no certain diagnosis can be made, unless some of the encephaloid matter should be ejected during the softening and destruction of the tumour—which is not a very common occurrence, the patient often dying before the morbid growth has arrived at this stage.

"The symptoms which most unequivocally indicate Fungus or Scirrhus about the Cardia are, uneasiness there, impediment and pain in the deglutition of food, as if it rested or stuck at that point, and a ropy secretion from the fauces, with occasional nausea, and retching. Regurgitation of solids or liquids is not unfrequent during or after meals. In the course of either complaint, the surface becomes of a sallow, sickly hue, and has a withered aspect and feel; while the flesh wastes in proportion to the progress of the disease, the skin at last becoming dry, loose, and wrinkled, through the absorption of fat from the subjacent cellular membrane. It may be here remarked, that less emaciation generally attends Fungus than Scirrhus, supposing each exist separately and independently of the other. This circumstance may proceed partly, perhaps, from the more rapid progress of Fungus; and when this state supervenes or attends Scirrhus, the case usually terminates more speedily than when Scirrhus occurs alone.

"The matter ejected from the Œsophagus in the first stage of Scirrhus very often closely resembles a mixture of arrow root in hot water, and even, when softening takes place, it frequently has a similar appearance; but where ulceration advances, it is apt, occasionally, to be blended with a bloody or ichorous discharge, or with coagulated blood itself, while lancinating pains succeed, great distress, fever, colliquative sweats, and much emaciation." 63.

### Stricture about the Pylorus from the above Diseases.

The symptoms of this dangerous malady are extremely obscure for some time, especially when the disease is schirrhus, for then they form more insidiously, and advance more slowly, than when fungus is seated in that part of the stomach.

"One of the first signs is a feeling of uneasiness in the direction of the Pylorus, which is the most apt to be felt after bodily fatigue, during anxiety, or after an unusually full or indigestible meal. The common train of dyspeptic suffering follows, namely, flatulence, irritation, sense of fullness or weight in the epigastric region, with depression of spirits or fretfulness of temper, and the complexion gradually acquires the cast of sickliness, and the patient loses flesh as well as colour. At length, on any extraordinary exertion, either of body or mind, nausea, retching, or vomiting unexpectedly takes place, and the quantity of fluid matter ejected frequently exceeds that which had been taken; but this irritability of the stomach is most liable to be displayed when, the mind being over-wrought, or the body fatigued, some solid or liquid material shall offend the stomach. At a further stage, the patient feels what he calls a forcing pain, or pain and pressure in the region of the Pylorus, as if the contents of the stomach met with a resistance at that point, when the organ acted to propel them into the intestines. This symptom generally occurs an hour or two after a meal, and is one of the most certain indications of Stricture of the Pylorus, increasing as the disease advances, together with the irritability of the stomach, and the loss of flesh and strength. In my own practice, I have only met with one exception to this remark, and in that case the patient never complained of urgent pain, never vomited in the course of the disease, but then he instinctively took extremely small quantities of food at once, and that of the blandest description. Solid animal food commonly excites the most uneasiness, and is often followed by eructations of an offensive taste, rolling of wind as if confined in the stomach, with a sense of rising or choaking in the throat. On some occasions I have known macerated shreds of mutton or beef brought up several days after a meal of one or the other. Lassitude and languor increase in such examples, and the heart generally becoming much attenuated, the pulse is very feeble, and sometimes the patient is liable to feelings of faintness, more especially on exertion of the body. In Scirrhus, the matter which comes from the Stomach is generally like arrow root mixed with hot water, or ropy like an infusion of lint seed, whereas in Fungus it commonly has a more curdly character—an appearance which may sometimes assist in the discrimination of the one state from the other; but in both the ejection of a glairy or grumous liquid may considerably exceed the quantity of fluid which had been drunk. Sometimes however, Fungus, but particularly Scirrhus, may exist, about the Pylorus or central portions of the Stomach for two or three months before vomiting takes place—a fact which should be remembered in the investigation of the seat of such affections." 64.

Dr. A. observes that, in all cases of strictured pylorus he found great emaciation, but that the case of Buonaparte was an exception. We believe Napoleon's stomach was generally affected with scirrhus, but that there was no particular stricture of the pylorus. Amongst the causes of this dreadful disease, Dr. A. properly ranks mental anxiety.

"Most of the ancient philosophers lived to an extreme old age, their pursuits being so benevolent and their thoughts so innocent as necessarily to lead to mental serenity; but Napoleon was not a philosopher, and though he attempted to govern the whole civilized world, had so little command over himself, that he allowed his mind to be incessantly disturbed even by little things, especially towards the close of his career, and thus, probably, at last converted an hereditary tendency into a destructive disease." 65.

### Fungus or Scirrhus of the Stomach between the Cardia and Pylorus.

It has been remarked, that when the stomach is universally affected with organic disease, there is often less functional derangement than when a small portion only is affected. Dr. A. is inclined to agree in this remark. This, indeed, was the case with Napoleon. The real disease was not suspected before death—as the liver was the organ supposed to be diseased.

"When Fungus or Scirrhus attacks the central parts of the Stomach, the most pathognomonic Symptom is pain after any thing has been eaten or drunk, the history of the case not corresponding to that of Simple Inflammation of the Mucous texture, in other respects. For instance, the tongue is not so preternaturally red in general, but surprisingly natural for the most part, considering the permanency of the character, and the progressively deep impressions of the disease. The appetite too, is, in general, not prostrate as in Chronic Muco-Gastritis, for, on the contrary, the patient usually longs for this or that kind of food, but his taste is so capricious, that if the practitioner regulated it to-day, the restriction is sure to be violated to-morrow, or shortly afterwards. The pulse is not above par in power or in frequency, as in Chronic Muco-Gastritis, for a considerable period from the accession of the complaint: indeed, in the majority of examples it only becomes so on the solution or breaking up of Fungus or Scirrhus, when it frequently happens, that the circulation is disturbed by a sort of febrile commotion. From first to last there is a wasting of the flesh, and a loss of colour, with occasional attacks of nausea, retching, or vomiting, particularly on exercise, over-exertion, or indulgence of appetite; and now and then a tenacious fluid is thrown from the Stomach, which, in the last stage of softening, is usually mixed with a dark, acrid, or offensive matter, as before described. An examination of the epigastrium, by the fingers, may sometimes detect hardness and swelling, so as to lead to the presumption, that organic disease exists in the Stomach; but such irregularities, even when present, are not always tangible through the integuments, and therefore we should rather rely, conjointly, upon the past history, and the present concourse of uneasy feelings, and interrupted functions, with reference to the Stomach. Upon the whole, I should say, from my own experience, that more pain generally attends Scirrhus than Fungus of this part, particularly towards the conclusion; and at that time a sensation of frequent faintness is not uncommon, with a tumultuous feeling or oppression about the heart, as often happens in affections of the Pylorus. In almost all great organic derangements of this nature seated in the Stomach, a distinct and even strong pulsation is felt in the epigastrium, a symptom which, perhaps, may arise from the pressure of the morbid mass upon the cœliac artery or abdominal aorta; but whatever may be the explanation of this sign, it will be found one of the most constant in the progress or towards the conclusion of Scirrhus or Fungus of the Stomach, and wherever it exists, in conjunction with a sallow surface, irritability, or other uneasiness of the Stomach, and loss of flesh, Fungus or Scirrhus of that organ may be fairly suspected." 66.

The difficulty of recognizing the existence of these diseases is sometimes sufficiently obvious—but the distinction of scirrhus from malignant fungus we believe to be little more than guess-work. Fortunately it is not of very vital consequence, as to treatment. With the following extract, the work, and our analysis of it, conclude.

"There is something forcibly striking in the expression of the countenance and colour of the skin in most great organic diseases. Thus, in Tubercular disease of the lungs and elsewhere, the lucid cornea becomes more glary and the conjunctiva more blanched and pearly than natural, with a softness and almost pensiveness of expression; while the face grows more and more sharp, and the surface acquires a much more delicate hue, as is exemplified in the hands, which are then remarkably white, the prominent veins wandering across them, like fine blue lines through pure marble. Again, the expression in Scirrhus considerably advanced, is that of more or less solicitude, and the skin commonly has a sallow tint, like that of the pale yellowish willow; whereas, the cast of the countenance is less materially affected in Fungus, and the skin has neither the delicacy attendant on Tubercle, nor the sallowness attendant on Scirrhus, but is often of a dull muddled white, almost resembling that of common tallow or painters' putty. It is indeed difficult, nay impossible, to convey in words very precise descriptions of the varieties of expression and colour; but there is, nevertheless, some change about the face and skin which often, at first sight, leads an experienced eye to suspect the presence of deep visceral derangement. Yet every prudent man will not be guided by first impressions, but pause and accurately investigate all the particulars of the case, and then deliberately reflect upon them, in order to legitimately deduce a correct opinion. The intelligent pathologist will be especially on his guard not to confound the ventricular disturbance and sallowish aspect of the sedentary and studious dyspeptic with any organic disease of the Stomach; for, though in such persons the face be 'sicklied o'er with the pale cast of thought,' yet it is most frequently an indication merely of a disorder, which admits of a ready remedy, and which, even if continued under the same character, may not at all shorten life." 68.

The plates in this fasciculus are five in number, beautifully executed, and highly expresive of the scirrhous, fungoid, and ulcerative diseases, that affect the œsophagus, cardia, gastric parietes, and pylorus. Doubtless the colouring is, in most of the plates, much too high; but, as we have often said before, this imperfection appears to be inseparable from pathological drawings. The uncoloured plates are, in fact, much more faithful than those which are embellished by the pencil; so that it may be truly said of them, that when unadorned, they are adorned the most. It does not appear that the treatment of these dreadful diseases came within the scope of the work, as nothing is said of it in the second fasciculus. But what, in truth, can be said on such a hopeless subject? It is of great consequence, however, to be able to say whether there is, or is not, organic disease in any given case. The dangerous process of POLYPHARMACY may thus be deprived of its horrors, and the remedial art be prevented from becoming the instrument of destruction. We hope Dr. Armstrong will persevere in portraying the organic maladies to which flesh is heir, in a style which does infinite honour to the pencil of Mr. Cox, and the lithographic press of Mr. Fairland.

